# Pulmonary Oxidative Stress, Inflammation and Cancer: Respirable Particulate Matter, Fibrous Dusts and Ozone as Major Causes of Lung Carcinogenesis through Reactive Oxygen Species Mechanisms

**DOI:** 10.3390/ijerph10093886

**Published:** 2013-08-27

**Authors:** Athanasios Valavanidis, Thomais Vlachogianni, Konstantinos Fiotakis, Spyridon Loridas

**Affiliations:** Department of Chemistry, University of Athens, University Campus Zografou, Athens 15784, Greece; E-Mails: thvlach@chem.uoa.gr (T.V.); cfiot@chem.uoa.gr (K.F.); spiroslor@chem.uoa.gr (S.L.)

**Keywords:** reactive oxygen species, oxidative stress, inflammation, mechanisms of carcinogenesis, respirable particulate matter, ozone, tobacco smoke

## Abstract

Reactive oxygen or nitrogen species (ROS, RNS) and oxidative stress in the respiratory system increase the production of mediators of pulmonary inflammation and initiate or promote mechanisms of carcinogenesis. The lungs are exposed daily to oxidants generated either endogenously or exogenously (air pollutants, cigarette smoke, *etc.*). Cells in aerobic organisms are protected against oxidative damage by enzymatic and non-enzymatic antioxidant systems. Recent epidemiologic investigations have shown associations between increased incidence of respiratory diseases and lung cancer from exposure to low levels of various forms of respirable fibers and particulate matter (PM), at occupational or urban air polluting environments. Lung cancer increases substantially for tobacco smokers due to the synergistic effects in the generation of ROS, leading to oxidative stress and inflammation with high DNA damage potential. Physical and chemical characteristics of particles (size, transition metal content, speciation, stable free radicals, *etc.*) play an important role in oxidative stress. In turn, oxidative stress initiates the synthesis of mediators of pulmonary inflammation in lung epithelial cells and initiation of carcinogenic mechanisms. Inhalable quartz, metal powders, mineral asbestos fibers, ozone, soot from gasoline and diesel engines, tobacco smoke and PM from ambient air pollution (PM_10_ and PM_2.5_) are involved in various oxidative stress mechanisms. Pulmonary cancer initiation and promotion has been linked to a series of biochemical pathways of oxidative stress, DNA oxidative damage, macrophage stimulation, telomere shortening, modulation of gene expression and activation of transcription factors with important role in carcinogenesis. In this review we are presenting the role of ROS and oxidative stress in the production of mediators of pulmonary inflammation and mechanisms of carcinogenesis.

## 1. Introduction

Studies of pulmonary carcinogenicity and respiratory inflammation have shown high risk for respiratory diseases and lung carcinogenesis in humans from exposures to various inhalable dusts, mineral fibers, airborne particulate matter (PM) and ozone. Ambient air pollution, containing PM smaller than aerodynamic diameter of 2.5 μm (PM_2.5_), has gained particular attention in recent years as a causative factor in the increased incidence of respiratory diseases, including lung cancer [1,2,3,4]. Tobacco smoke also plays a very important role in increasing the risk for epithelial inflammation and lung cancer due to its high carcinogenic potential and the synergistic effects with other respirable particulate to generate reactive oxygen species (ROS) and catalyze redox reactions in human lung epithelial cells, leading to oxidative stress and increased production of mediators of pulmonary inflammation [5,6,7,8,9].

Airborne PM is an important pollutant of urban atmosphere and has been linked to adverse health effects of the respiratory system. Particles can penetrate into the respiratory airways and deposited in the respiratory bronchioles and the alveoli. PM from combustion sources contain a number of constituents that generate ROS by a variety of reactions. The most important are transition metals with redox properties, persistent free radicals, redox-cycling of quinones, polycyclic aromatic hydrocarbons (PAHs) and volatile organic compounds (VOCs) which may be metabolically activated to ROS that can react to form bulky adducts or strand breaks on cellular DNA [10,11].

In recent years a number of publications have linked air pollution particle exposure to oxidation of DNA in cells, tissues, or their metabolites in urine of rodents and humans. Studies have investigated the effect of diesel exhaust particles (DEP) or ambient air pollutants in terms of oxidized DNA nucleobases. In nuclear and mitochondrial DNA (mtDNA) a free radical-induced oxidative lesion has been used widely as a predominant biomarker for oxidative stress. 8-Oxo-7,8-dihydroguanine (8-oxoGua) can be measured quantitatively by HPLC and GC-MS in animal and in human biomonitoring studies [12,13,14,15].

Additionally, other air pollutants contribute to oxidative stress in the pulmonary system and play a role in synergistic redox effects. Ozone as a pulmonary irritant causes oxidative stress, inflammation and tissue injury. Also, evidence suggests that macrophages play a role in the pathogenic pulmonary responses. Studies showed that bronchiolar epithelium is highly susceptible to injury and oxidative stress induced by acute exposure to ozone, accompanied by altered lung functioning [16]. Another study showed that exposure of rats to ozone resulted in increased expression of 8-hydroxy-2′-deoxyguanosine (8-OHdG), as well as heme oxygenase-1 (HO-1) in alveolar macrophages [17]. Epidemiological and clinical studies showed that people exposed to combined air pollutants, such as ozone and cigarette smoke or PM showed increased pulmonary oxidative stress and inflammation associated with an increase in pulmonary diseases and mortality [18].

## 2. Why Aerobic Organisms are Susceptible to Oxidative Stress, Inflammation and Degenerative Diseases?

All aerobic biological systems use oxygen as an essential part of their physiological cellular metabolic processes. At moderate concentrations oxygen free radicals, or more generally, reactive oxygen species (ROS) and reactive nitrogen species (RNS), are products of normal cellular metabolism. Aerobic organisms generates energy in the mitochondria of eukaryotic cells and most of these ROS and RNS physiological and beneficial; however, less than 5% of them can be toxic for the cell if their concentration increases. These normally low-concentration compounds that are derived from oxidative metabolism are necessary for certain subcellular events, including signal transduction, enzyme activation, gene expression, *etc.* [19,20].

ROS are produced in cellular sites by electron transfer reactions through enzymatic and non-enzymatic processes. The main sources of ROS in all cells of aerobic organisms are mitochondria, cytochrome P450 and peroxisomes. Under physiological conditions, there is a constant endogenous production of reactive intermediates of radical species of oxygen and nitrogen that interact as regulatory mediators of signaling processes for metabolism, cell cycle, intercellular transduction pathways, cellular redox systems and mechanisms of apoptosis [19,20,21,22,23]. At higher concentrations, free radicals and ROS are hazardous for living organisms and can cause oxidative damage to all major cellular constituents (membrane lipids, proteins, enzymes, DNA). Many of the ROS-mediated responses actually protect the cells against oxidative stress and re-establish “redox homeostasis”. Excessive or sustained increase in ROS production, which if not balanced by antioxidant enzymes and non-enzymatic cellular antioxidant defences, has been implicated in chronic inflammatory conditions, malignant neoplasms, diabetes mellitus, atherosclerosis and various neurodegenerative diseases [24,25,26,27,28].

Reactive organic species in aerobic organisms can be classified into four groups: ROS, reactive nitrogen species (RNS), reactive sulfur species (RSS) and reactive chlorine species (RCS). ROS are the most abundantly produced and have highly oxidative properties which can damage essential cellular components. If ROS and the other reactive species are not balanced by antioxidant enzymes and non enzymatic antioxidant substances with low molecular mass. The most important ROS include superoxide anion (O_2_^−^·), hydrogen peroxide (H_2_O_2_), the most reactive hydroxyl radical (HO·), singlet oxygen (^1^O_2_), ozone (O_3_) and others. The most abundant RNS is nitric oxide (NO·), which is able to react with certain ROS, including the peroxynitrite anion (ONOO^−^, interaction of O_2_^−^· and NO·). Nitric oxide can be converted into peroxynitrous acid and ultimately into hydroxyl radical and nitrite anion (NO_2_^−^) [29]. All aerobic organisms evolved antioxidant defences to protect themselves against ROS generated *in vivo*. These antioxidants can intercept, scavenge, neutralize radicals and reactive intermediates generated in excess under physiological conditions. The most important antioxidant enzymes are the superoxide dismutase (SOD), the catalase (CAT), the glutathione redox system (glutathione peroxidase and glurtathione-S-transferase) [30,31]. Also, low molecular mass antioxidant compounds are ascorbic acid (vitamin C), vitamin E (tocopherol), uric acid, bilirubin, glucose, caeruloplasmin, *etc.*, and proteins that bind metal ions in forms unable to accelerate free radical reactions. These defences are very useful in the protection of aerobic organisms from oxidative damage, such as lipid peroxidation, protein or enzyme oxidation and DNA oxidative damage (mutations) [32].

Oxidative stress results when ROS or RNS are not adequately removed or neutralized, because of depleted antioxidant defences of increases beyond their capacity. The balance between oxidants and antioxidants “redox homeostasis”, is a crucial event in living organisms. Subjecting cells to oxidative stress can result in severe metabolic dysfunction and other oxidative damages to proteins, carbohydrates, DNA, RNA, mtDNA and membrane lipids [33,34,35].

Carcinogenesis is a multistep process in three stages: initiation, promotion and progression leading to malignant tumours [36,37]. During these stages various genetic and epigenetic events occur that lead to the progressive conversion of normal cells into cancer cells. In cellular prooxidant states ROS are overproduced, thus initiating oxidative damage to cellular DNA (cDNA) and mitochondrial DNA. The biological consequences are mutations, chromosomal aberrations, sister chromatid exchanges, *etc.*, that lead to cellular degeneration, carcinogenesis and ageing. ROS play a role mostly in the promotion stage of carcinogenesis, during which gene expression of initiated cells is modulated by affecting genes that regulate cell differentiation and growth. In the progression stage, benign neoplasms are stimulated to more rapid growth and malignancy [38,39].

## 3. Oxidative Stress, Inflammation and Carcinogenesis

Oxidative damage is considered to play a pivotal role in ageing, several degenerative diseases and cancer. Acute and chronic inflammation has been correlated with increased risk for various malignant neoplasms from a great number of clinical and other studies. The possible mechanisms by which inflammation can contribute to carcinogenesis include genomic instability, alterations in epigenetic events and subsequent inappropriate gene expression, enhanced proliferation of initiated cells, resistance to apoptosis, aggressive tumour neovascularization, invasion through tumour-associated basement membrane, angiogenesis and metastasis [40,41,42]. Inflammatory cells are particularly effective in generating most of the reactive oxygen species. The activation of the redox metabolism of the inflammatory cells generates a highly oxidative environment within an organ of aerobic organisms. Much of the oxygen biochemistry, through the activation of plasma membrane NADPH oxidase, of macrophages and neutrophils is directed towards the release of superoxide anion, hydrogen peroxide and hydroxyl radicals [43,44,45,46,47,48].

Inflammation acts through the formation of ROS and RNS which cause oxidative damage to cellular components. Many proinflammatory mediators, especially cytokines, chemokines and prostagladins, turn on the angiogenesis switches mainly controlled by vascular endothelial growth factors [49,50].

Cancer-associated inflammation is also linked with immune-suppression that allows cancer cells to evade detection by the immune system. Inflammation is a critical component of tumour progression. Many cancers arise from sites of infection, chronic irritation and inflammation. It is now becoming clear that the tumour microenvironment, which is largely orchestrated by inflammatory cells, is an indispensable participant in the neoplastic process, fostering proliferation, survival and migration. In addition, tumour cells have co-opted some of the signalling molecules of the innate immune system, such as selectins, chemokines and their receptors for invasion, migration and metastasis. These insights are fostering new anti-inflammatory therapeutic approaches to cancer development [51]. Pathological angiogenesis is a hallmark of cancer and various ischaemic and inflammatory diseases. Chronic inflammation is associated with angiogenesis, a process that helps cancer cells to grow. Angiogenesis is necessary for expansion of tumor mass. Macrophage, platelets, fibroblasts and tumour cells are a major source of angiogenic factors (vascular endothelia growth factors, chemokines, NO, *etc.*) [52,53,54]. 

The inflammation in the tumour microenvironment is characterized by leukocyte infiltration, ranging in size, distribution and composition, as: tumor-associated macrophages (TAM), mast cells, dendritic cells, natural killer (NK) cells, neutrophils, eosinophils and lymphocytes. These cells produce a variety of cytotoxic mediators such as ROS and RNS, serine and cysteine proteases, membrane perforating agents, matrix metalloproteinase (MMP), tumour necrosis factor α (TNFα), interleukins (IL-1, IL-6, IL-8), interferons (IFNs) and enzymes, as cyclooxygenase-2 (COX-2), lipooxygenase-5 (LOX-5) and phospholipase A2 (PLA2). Other researchers discovered that tumour-associated macrophages are key regulators of the link between inflammation and carcinogenesis [55,56].

## 4. Particulate Matter, ROS, Oxidative Stress and Inflammation

The main current paradigm in respirable particular matter toxicology is centred on the concept of oxidative stress. The ability of respirable particles or fibrous dusts to penetrate the respiratory system and reach the lung alveoli in order to generate ROS and other oxidants or free radicals is suggested to be the main factor involved in their pathogenic potential. There is abundance of scientific evidence for ROS involvement in lipid peroxidation, DNA mutations and protein (enzyme) oxidative damage [57,58,59].

The term “oxidative stress” is defined as the adverse condition resulting from an imbalance in cellular oxidants (mostly ROS and nitrogen free radicals) and antioxidants (enzymatic, such as CAT and SOD, and small molecular compounds with antioxidant properties, such as ascorbic acid). But small amounts of ROS and RNS are similarly very important for various biochemical processes and intracellular signaling [60]. Redox homeostasis is very important for aerobic organisms. It is governed by the presence of large pools of these antioxidants that absorb and buffer reductants and oxidants. Most importantly, antioxidants provide essential information on cellular redox state, and they influence gene expression associated with biotic and abiotic stress responses to maximize defense. Growing evidence suggests a model for redox homeostasis in which the ROS–antioxidant interaction acts as a metabolic interface for signals derived from metabolism and from the environment. This interface modulates the appropriate induction of acclimation processes or, alternatively, execution of cell death programs [61]. Oxidative stress is the responsible factor for the rise of pulmonary pathology through airway inflammation, particularly when humans are exposed to inhalable airborne PM [62,63].

Experimental studies showed that airborne PM, like tobacco smoke, produce ROS that have been implicated in the activation of mitogen-activated protein kinase (MAPK) family members and activation of transcription factors such as NF-κB and AP-1 (the activator protein 1). These signaling pathways have been implicated in processes of inflammation, apoptosis, proliferation, transformation and differentiation [64]. Airborne PM represents a mixture of many different components that consists of a variable particle core and a large array of surface-bound constituents including PAHs and heavy metals. Also, studies showed that stable free radicals in the PM, as well as in cigarette tar play an important role in increasing ROS production, especially hydroxyl radicals [65,66,67].

The most important ROS is considered the hydroxyl radicals (HO·) via Fenton reaction where metals can be oxidized by hydrogen peroxide (Fe^2+^ + H_2_O_2_ → Fe^3+^ + HO^−^ + HO·). It is very reactive and is widely considered to be responsible for damage to DNA and lipid peroxidations [68,69]. Studies showed that airborne particulates have absorbed numerous reactive organic compounds on their porous surface which can release ROS via quinone redox cycling [70]. PAHs can be converted to quinones within the lung tissues by biotransformation enzymes such as cytochrome P450, epoxide hydrolase and dihydrodiol dehydrogenase [71,72].

Synergistic mechanisms of inhalable particulate matter (penetrating deep into the lung’s alveoli) and other components of air pollution (ozone, nitric oxide, soot, heavy metals, PAHs) and tobacco smoke have been studied. The porous surfaces of airborne particles provide a fertile ground for catalyzing the increased generation of ROS or other damaging oxidants which are potential initiators of pulmonary carcinogenesis. Synergistic effects for increased ROS formation have been experimentally verified [72].

Synergistic effects for increasing ROS generation has been verified by various experimental studies. Increasing ROS generation has been observed: between ambient transition metals and PM quinoids [73], for air pollution PAHs and tobacco smoke [74], for cigarette tar and nitric oxide [75], between soot (impure carbon particles from the incomplete combustion) and iron particles [76], between components of air pollution with aerodynamic diameter PM_10_ [77], between airborne PM and ozone [78,79], between tobacco smoke and PM stable free radicals [80], between mineral fibes and tobacco smoke [81] and between ambient air MP and wood smoke PM [82].

Aside from direct generation by the airborne particles and other inhalable materials, ROS can be generated by target cells such as lung epithelial cells and pulmonary macrophages upon interaction with and/or uptake of particulate material. Phagocytic cells of the innate immune system such as alveolar macrophages (AM) and polymorphonuclear neutrophils (PMN) are highly proficient producers of ROS to enhance microbicidal conditions in phagocytic vacuoles and eliminate pathogenic bacteria or potentially harmful particles [83,84]. Resident macrophages in the airways and the alveolar spaces can release ROS/RNS after phagocytosis of inhaled particles. These macrophages also release large amounts of TNF-alpha), a cytokine that can generate responses within the airway epithelium dependent upon intracellular generation of ROS/RNS. As a result, signal transduction pathways are set in motion that may contribute to inflammation and other pathobiological damage in the lung airways. Such effects include increased expression of intercellular adhesion molecule 1, interleukin-6, cytosolic and inducible nitric oxide synthase, manganese superoxide dismutase (MnSOD), cytosolic phospholipase A2, and hypersecretion of mucus. Ultimately, ROS/RNS may play a role in the global response of the airway epithelium to particulate pollutants via activation of kinases and transcription factors common to many response genes. Thus, defense mechanisms involved in responding to offending particulates may result in a complex cascade of events that can contribute to airway pathology [85].

Phagocyte-generated reactive species include superoxide anion (O_2_^−^·), nitric oxide, free radical (NO·), hydrogen peroxide (H_2_O_2_), the extremely reactive hydroxyl radical (HO·), the highly oxidant peroxynitrite (ONOO^−^) and hypochlorous acid (HOCl) [86]. Additionally, myeloperoxidase (MPO) can produce the highly toxic product HOCl from H_2_O_2_ and chloride anions. Another phagocyte anti-microbial system is the enzyme inducible nitric oxide synthase (iNOS), which generates NO· radicals from l-Arginine, NADPH and oxygen. The principal cellular source of NO· in the lung are the macrophages, with neutrophils representing another source. Also, as iNOS is expressed in airway epithelium, lung epithelial cells release NO· in response to inflammatory stimuli such as endotoxin and cytokines. Superoxide anion formed by NADPH oxidase (NOX) can react with NO· formed by iNOS to produce the highly oxidant peroxynitrite ONOO^−^ [87].

Although ROS and RNS production is a crucial event in host defence, they are capable of causing damage to cellular macromolecules such as nucleic acids, lipids and proteins. In a chronic inflammatory setting, persistent production of ROS can cause considerable tissue damage. Therefore, people suffering from chronic inflammatory diseases, especially those localised to or affecting the pulmonary region, may be susceptible for adverse health effects of PM inhalation [88]. It is well known that ROS/RNS are capable of causing DNA strand breaks and DNA oxidation, factors that are implicated in the initiation stage of carcinogenesis. One of the major products of oxidative DNA damage is the premutagenic lesion 8-hydroxy-2′-deoxyguanosine (8-OHdG). Several *in vitro* studies have demonstrated that PM induces DNA damage in the form of strand breaks and 8-OHdG formation in lung epithelial cells [89,90]. Ambient particulate matter has been the focus of numerous toxicological studies and the generation of ROS in pulmonary cells is considered the most important mechanism for carcinogenesis [91].

## 5. Endogenous and Exogenous ROS, Lipid Peroxidation and DNA Damage

Endogenous and exogenous oxidative damage to DNA of aerobic cells from attack by ROS is very frequent and is accepted after numerous measurements as an experimental fact. It has been estimated that one human cell is exposed daily to approximate 10^3^–10^5^ hits by HO· alone. The highly reactive HO· reacts with the heterocyclic DNA nucleobases and the sugar moiety near or at diffusion-controlled rates leading to adduct radicals [92,93,94,95].

The mitochondrial DNA is very gene dense and encodes factors critical for oxidative phosphorylation but it is exposed to greater oxidative damage due to the mistakes that occur during the production of ATP through the electron transport chain Mutations of mtDNA cause a variety of human mitochondrial diseases and are also heavily implicated in age-associated diseases, such as cancer, and aging. There has been considerable progress in understanding the role of mtDNA mutations in human pathology during the last two decades, but important mechanisms in mitochondrial genetics remain to be explained at the molecular level. In addition, mounting evidence suggests that most mtDNA mutations may be generated by replication errors and not by accumulated damage [96].

Analytical measurements of the oxidative DNA damage, as the adduct 8-oxo-2′-deoxyguanosine (8-oxodG) in mitochondrial DNA (mtDNA), proved that it suffers greater endogenous oxidative damage than nuclear DNA (cDNA) because of its proximity to the mitochondrial electron transport chain, lack of protective histones and less efficient repair mechanisms [97,98,99]. DNA damage and increasing numbers of mutations driving the malignant transformation process. Recent evidence also indicates that the resulting mutated cancer-causing proteins feedback to amplify the process by directly affecting mitochondrial function in combinatorial ways that intersect to play a major role in promoting a vicious spiral of malignant cell transformation. Consequently, many malignant processes involve periods of increased mitochondrial ROS production when a few cells survive the more common process of oxidative damage induced cell senescence and finally death [100].

Airborne PM generated from combustion of fuels have a high potential for the formation of ROS that deplete endogenous antioxidants, alter mitochondrial function and produce oxidative damage to lipids, enzymes and DNA. Surface area, redox reactivity and chemical composition play important roles in the oxidative potential of particulates. Biomonitoring studies in humans have shown associations between exposure to air pollution and wood smoke particulates and high rates of oxidative damage to DNA [101]. According to experimental studies on the elucidation of mechanisms by oxidative DNA damage contributes to the development of carcinogenesis, there are at least two different mechanisms thought to play an important role. The first mechanism is thought to act through the modulation of gene expression affecting intracellular signaling pathways, whereas the second is thought to proceed through the induction of genetic damage (mutations, strand breaks, chromosomal rearrangements) and a blockage to the DNA replication [102,103].

Studies showed that modulation of gene expression by ROS and RNS and the resulting oxidative stress is an important mechanism of carcinogenesis. Epigenetic effects on gene expression can lead to the simulation of growth signals and proliferation. Oxidative stress causes strand breakage of DNA and incomplete enzymatic repair mechanisms that lead to chromosomal changes. These changes in turn contribute to genetic amplification, alterations in gene expression and loss of heterozygosity, resulting in the promotion of mechanisms of progression of carcinogenesis [104,105,106].

Direct evidence of the generation of ROS, activation of redox sensitive transcription factors (NF-kappaB, AP-1 and Nrf2) and alteration of ROS-related gene expression have been proved experimentally with various carcinogenic metals [Cd, As, Ni, Cr(VI)]. Transition metals have been measured in substantial concentrations in urban air pollution PM. In addition to oxidative DNA damage, metals cause changes in DNA methylation and histone modifications, leading to epigenetic silencing or reactivation of gene expression [107,108,109]. *In vivo* experiments showed that in iron-induced carcinogenesis there are target genes and oxidative DNA damage [110]. In the second mechanism ROS and RNS cause genetic mutations and chromosomal rearrangements [111].

One of the most important and widely studied oxidative DNA damage is the adduct of hydroxyl radicals on DNA nucleobases, especially deoxynucleosides, The 8-hydroxy-2′-deoxyguanosine (8-OHdG) and/or its tautomeric 8-oxo-7,8-dihydro-2′-deoxyguanosine (8-oxodG) have been proved important mutagenic adducts to DNA and therefore potential biomarkers of carcinogenesis. Mutations of 8-oxodG involve a GC → TA transversion [112,113]. Also, ROS and RNS with their oxidative potential may initiate carcinogenesis by attacking DNA nucleobases causing certain changes in oncogenes and tumour suppressor genes [114]. Experimental results showed that HO· generates DNA phenotypes with various metastatic potential that are likely to contribute to the diverse physiological properties and heterogeneity characteristic of metastatic cell population [115].

Clinical and epidemiological evidence from animal studies showed that consumption of animal fats, strongly influence the incidence of mammary and sigmoid colon cancers, through lipid peroxidation [116,117,118,119]. High fat diets cause proliferation of rat hepatic peroxisomes and an increase of peroxisomal and mitochondrial enzymes involved in fatty acid β-oxidation [120]. These changes and other fat metabolites, such as unsaturated aldehydes, may play a critical role in oxidative DNA damage and the liberation of low levels of H_2_O_2_, supporting the suggestion that direct DNA damage may be the mode of carcinogenic action of peroxisome proliferators [121,122]. Lipid peroxidation generates a complex variety of reactive electrophilic products and free radicals, that react with proteins and DNA [123,124].

ROS and RNS can react with cellular membrane phospholipids generating lipoperoxide radicals (LOO·) and toxic aldehydes (malondialdehyde, MDA) that can alter membrane permeability and microcirculation. Also, they can activate nuclear factors leading to other proi-nflammatory agents. Malondialdehyde is a naturally occurring product of lipid peroxidation and prostaglandin biosynthesis, which is mutagenic and carcinogenic compound. MDA reacts with DNA to form adducts to nucleobases deoxyguanosine and deoxyadenosine [125]. MDA was shown to be carcinogenic in mice and tumours arose in a very short time after administration [126]. Several other adlehydes, including α,β-unsaturated aldehydes have been proved mutagenic in *Salmonella typhimurium* [127,128]. Lipid peroxidation, inflammation produce MDA which reacts with nucleobases to form multiple adducts with mutagenic potential [129,130,131].

## 6. Telomere Shortening and Carcinogenesis. Epigenetic Effects Mediated by ROS. (Modulation of Gene Expression and Activation of Transcription Factors)

Telomere is a region of repetitive nucleotide sequences at each end of a chromatid protecting the end of the chromosome from deterioration or from fusion with neighboring chromosomes of most eukaryotic organisms, regulating many crucial cellular functions in multicellular organisms such as ageing and cancer [132]. Telomere repeats are most often maintained by a specialized reverse transcriptase called telomerase. The interplay among telomeric repeats, telomerase and telomere-binding proteins governs a wide range of cellular processes, including chromosomal stability, gene expression and cell replicative life span [133,134]. Telomere are sensitive to shortening due to oxidative DNA damage can be very important for carcinogenesis [135,136].

Damaged nucleobases due to ROS oxidative stress can accumulate over the life span contributing significantly to the senescence of an organism. It has been found that senescent cells contain 30% more oxidatively modified guanines in their DNA and four times more 8-oxodG bases, a well studied biomarker of oxidative damage by ROS produced mainly from exposure to mineral fibres, dusts, particulate matter and heavy metals [137,138]. When human telomeres become very short they are no longer able to protect chromosome ends, particularly in the setting of loss of function of the retinoblastoma (pRB)) and p53 tumour-suppressor pathways. The resulting genomic instability leads to chromosomal breaks and fusion that permit the acquisition of further genetic changes and mutations. Genome instability is known as a hallmark of most human cancers. Scientists were arguing that collapse in telomere function can explain a significant portion of genetic instability in tumours [139,140,141]. Recent findings suggest that telomere maintenance might not be an obligate requirement for initial tumour formation in some settings and that telomerase (reverse transcriptase that provides the template for the telomere synthesis reaction) activation contributes to tumour formation independently of its role in maintaining telomere length [142].

Ageing is known from various studies that increases the susceptibility of tissues to carcinogenesis by several mechanisms: (i) tissue accumulation of cells in late stage of carcinogenesis; (ii) alterations in homeostasis in immune and endocrine systems; (iii) telomere instability because of oxidative shortening linking ageing with increased cancer risk. There is strong experimental evidence supporting the relevance of replicative senescence of human cells and telomere biology to human cancer [143]. Studies showed that exposure to airborne PM (in urban settings) and tobacco smoke are important factors in telomere shortening (or erosion) in humans [144,145,146].

The epigenetic effects caused by ROS and RNS, especially the adaptive responses induced in mammalian cells by the upregulation of stress-response genes, encode enzymatic antioxidant defence mechanisms. Low or transient ROS concentrations increase cell proliferation, through altered expression of growth factors and proto-oncogenes, whereas high ROS production lead to induction of apoptosis. ROS can induce alteration of gene expression that can occur through modulation of a host of signaling pathways, such as cAMP-mediated cascades, calcium-calmodulin pathways and intracellular signal transducers of nitric oxide (NO·) [147].

ROS and other oxidants can stimulate signal transduction pathways and lead to activation of key transcription factors such as Nrf2 and NF-κB. The resultant altered gene expression patterns evoked can contribute to the carcinogenesis process. Recent evidence demonstrates an association between a number of single nucleotide polymorphisms (SNPs) in oxidative DNA repair genes and antioxidant genes with human cancer susceptibility. These pathways regulate cellular migration, death, proliferation and survival, thus aberrant activation can paly a role in potential mechanisms of carcinogenesis [148]. Changes in epigenetic processes, such DNA methylation that leads to gene silencing without altering the DNA sequence, occur with air pollutant exposure, especially by global and gene-specific methylation changes. Respiratory cell line and animal models demonstrate distinct gene expression signatures in the transcriptome, arising from exposure to particulate matter or ozone. Particulate matter and other environmental pollutants alter expression of microRNA, which are short non-coding RNA that regulate gene expression [149]. Ageing also affects the epigenetic processes and mechanisms of carcinogenesis as well the oncogene expression and inevitably the spread of cancer and metastasis [150,151].

Careful study of genomic responses will improve the scientific understanding of mechanisms of lung injury from air pollution and will enable future clinical testing of interventions against toxic effects of air pollutants. Workplace exposures, such as tobacco smoke, particulates, diesel exhaust, polyaromatic hydrocarbons, ozone, and endotoxin, among others, enhance expression of inflammatory cytokines and allergic phenotypes by epigenetic means [152]. The transcription factors that are activated have been under the influence of cellular oxidants. ROS appear to influence selective activation of these transcription factors and in this way may explain the effects on cell death or cell proliferation in mechanisms of carcinogenesis, through exposure to ROS and oxidative stress. In this respect, scientists believe that inhibition of transcription factors must be targets for new anticancer drugs [153].

The most important transcription factors, correlated to activation by oxidants and implicated in the mechanisms of carcinogenesis are described briefly below. Most of these transcription factors are activated by particulate matter, tobacco smoke and mineral fibers that are generating ROS and other reactive oxidant species.

The *nuclear factor erythroid-derived 2 (Nrf2)*, which upon activation results in transcriptional expression of a broad spectrum of enzymes involved in xenobiotic detoxification, antioxidant response and proteome maintenance. Experiments with mice showed that they are more sensitive to the hepatic, pulmonary, ovarian, and neurotoxic consequences of acute exposures to environmental pollutants, inflammatory stresses and chronic exposures to cigarette smoke and other carcinogens [154]. Studies showed that the Nrf2 antioxidant and cellular detoxification program represents a previously unappreciated mediator of oncogenesis [155]. This factor has dual roles in carcinogenesis. In response to oxidative stress, the transcription factor NF-E2-related factor 2 (Nrf2) controls the fate of cells through transcriptional upregulation of antioxidant response element-bearing genes, including those encoding endogenous antioxidants, phase II detoxifying enzymes, and transporters. Expression of the Nrf2-dependent proteins is critical for ameliorating or eliminating toxicants/carcinogens to maintain cellular redox homeostasis [156].

The *activation protein-1 (AP-1)* contributes to basal gene expression in biological systems. ROS can activate AP-1 through several mechanisms. The effect of AP-1 activation is an increased cell proliferation due to increased expression of growth stimulatory genes such as cyclin D1. and suppression of the protein p21waf. Studies showed that AP-1 and NF-kappaB, inducible by tumour promoters of oxidative stimuli, show differential protein levels or activation in response to tumour promoters in JB6 cells. Researchers suggest that as long as oxidative events regulate AP-1 and NF-kappaB transactivation, these oxidative events can be important molecular targets for cancer prevention [157,158].

The *Nuclear Factor-kappaB (NF-kB*) (NF-kappa-light-chain enhancer of activated B cells), is expressed and participates in a wide range of biological processes, such as cell survival, differentiation, inflammation and growth [159]. NF-kB activation has been linked to a wide spectrum of extracellualr stimuli and oxidants and subsequent involvement in the carcinogenic process through promotion of angiogenesis and tumour cell invasion and metastasis [160,161].

Another transcription factor is *HIF-1 (a heterodimeric transcription factor*, *Hypoxia-inducible factor-1)* that has been implicated in ROS-induced carcinogenesis in a variety of human cancers [162,163]. The response to hypoxia in human cells is mainly regulated by hypoxia-inducible factors or HIFs which orchestrate signaling events leading to angiogenesis and tumorigenesis. A recent review underlined the molecular mechanisms for tumour hypoxia and their pro-tumour contribution to various biochemical changes leading to tumour cell metastasis [164].

Scientists experimented for years with the *p53*, that is a well known tumour suppressor gene. The p53 regulates cellular antioxidant defence and energy metabolism. The current theory suggest that neoplastic transformation consists of multistep accumulations of adverse genetic and epigenetic events. The p53 is a transcription factor that regulates cellular response to diverse forms of stress through a complex network which monitors genome integrity and cell homeostasis. It is generally accepted that p53 is a component in biochemical pathways central to human carcinogenesis. Study of p53 has come to the forefront of cancer research, and detection of its abnormalities during the development of tumors may have diagnostic, prognostic, and therapeutic implications [165]. A recent review described the tumor suppressor protein p53 which is susceptible to ROS and controls a wide variety of target genes and regulates numerous cellular functions in response to stresses that lead to genomic instability [166]. Another review discussed the multiple functions of p53 and how these correlate between cancer and neurodegeneration. Loss of function in p53 is usually associated with many common human cancers and p53 gene is mutated in almost half of all human cancers. There is a scientific hypothesis that the p53 conformational state is affected by ROS/RNS which may not be only a mere consequence of oxidative stress, but a fine gene transcription mechanism allowing specific adaptive responses. [167].

Recently, researchers discovered that glutaminase 2 (GLS2) is a p53 target. Phosphate-activated mitochondrial glutaminase (GLS2), and its expression is induced in response to DNA damage or oxidative stress in a p53 dependent manner. The GLS2 is a key enzyme in conversion of glutamine to glutamate and therefore a regulator of the synthesis of antioxidant glutathione (GSH). Increased concentration of GLS2 facilitates glutamide metabolism and lowers intracellular levels of ROS, resulting in an overall decrease in DNA damage as determined by 8-hydroxy-deoxyguanosine (8-OHdG) in both normal and stressed cells. This result provides evidence for a unique role for p53, linking glutamine metabolism, energy and ROS homeostasis, that may contribute to p53 tumor suppressor function [168].

## 7. Conclusions

In this review we presented the most important and recent studies concerning the role of ROS, RNS and other oxidants in the biochemical pathways of oxidative stress, pulmonary inflammation and the initiation of lung carcinogenesis. The human lungs are exposed continuously to air pollution oxidants in addition to endogenously generated ROS and RNS which are involved in physiological biochemical mechanisms and normal cellular signaling pathways. This review highlights the important role of inhalable particulate matter, mineral fibres, dusts, cigarette smoke and ozone in the generation of ROS, RNS, lipid radicals and other oxidants. In the last decade scientific evidence is available that supports the importance of oxidative stress and its correlation with increased incidence of malignant respiratory diseases due to inflammation, activation of transcriptional factors and DNA damage. Although all aerobic organisms are protected by enzymatic and non enzymatic antioxidant defences, an imbalance of prooxidants and antioxidants in the cellular environment can result to oxidative stress mechanisms, inflammation, carcinogenesis and ageing. During inflammation, enhanced ROS production induce DNA damage, inhibition of apoptosis, and activation of protooncogenes by initiating signal transduction pathways. Inflammatory cells are particularly effective in generating ROS and other reactive species, thus increasing oxidative damage and promoting mechanisms of carcinogenesis. Particularly DNA damage mediated by oxidative stress has been found by numerous studies to play an important role in the various stages of initiation, promotion and progression of cancer. Lipid peroxidation of cellular membranes and accumulation of mutagenic products in DNA can contribute to mechanisms of carcinogenesis, while telomere shortening through oxidative stress and senescence increases cancer risk.
